# Laparoscopic liver resection versus radiofrequency ablation for caudate lobe solitary hepatocellular carcinoma: A propensity score matching study

**DOI:** 10.1002/cam4.7068

**Published:** 2024-03-08

**Authors:** Zhengzheng Wang, Jingzhong Ouyang, Binyang Jia, Yanzhao Zhou, Yi Yang, Xiaoting Li, Qingjun Li, Jinxue Zhou

**Affiliations:** ^1^ Department of Hepatobiliary and Pancreatic Surgery The Affiliated Cancer Hospital of Zhengzhou University & Henan Cancer Hospital Zhengzhou China; ^2^ Department of Hepatobiliary Cancer, Liver Cancer Research Center Tianjin Medical University Cancer Institute and Hospital, National Clinical Research Center for Cancer, Key Laboratory of Cancer Prevention and Therapy, Tianjin's Clinical Research Center for Cancer Tianjin China; ^3^ Department of Interventional Therapy, National Cancer Center/National Clinical Research Center for Cancer/Cancer Hospital Chinese Academy of Medical Sciences and Peking Union Medical College Beijing China; ^4^ Department of Obstetrics and Gynecology, Henan Provincial People's Hospital People's Hospital of Zhengzhou University Zhengzhou China

**Keywords:** caudate lobe, hepatocellular carcinoma, laparoscopic liver resection, radiofrequency ablation

## Abstract

**Objective:**

This study aimed to compare the clinical efficacy of laparoscopic liver resection (LLR) and radiofrequency ablation (RFA) in treating solitary hepatocellular carcinoma (HCC) of the hepatic caudate lobe.

**Methods:**

Patients with hepatic caudate lobe HCC who underwent LLR or RFA at three hospitals in China between February 2015 and February 2021 were included. In total, 112 patients met the inclusion criteria, of whom 52 underwent RFA and 60 underwent LLR. The outcomes of the two groups were compared and analyzed using propensity score matching (PSM) method.

**Results:**

There were no significant differences between the two groups in terms of sex, HBV/HCV positivity, AFP positivity (>100 ng/mL), tumor position, Child–Pugh score, or preoperative liver function tests (ALT, AST, TBIL, ALB, and PT) (*p* > 0.05). Compared to the LLR group, the RFA group had a shorter operation time, less intraoperative bleeding, and shorter postoperative hospital stay (*p* < 0.05). There was no statistically significant difference in overall postoperative complications between the two groups (*p* > 0.05). Despite the larger tumor size, the LLR group had better postoperative recurrence‐free survival (RFS) (*p* = 0.00027) and overall survival (OS) (*p* = 0.0023) than the RFA group. After one‐to‐one PSM, 31 LLR patients and 31 RFA patients were selected for further analyses. The advantages of LLR over RFA were observed in terms of RFS (*p* < 0.0001) and OS (*p* = 0.00029).

**Conclusion:**

LLR should probably be recommended as the preferred method for solitary caudate lobe HCC.

## 1 INTRODUCTION

Hepatocellular carcinoma (HCC) is one of the most common solid malignant tumors in clinical practice worldwide and is the third leading cause of cancer‐related deaths.^1^ The incidence and mortality rates of HCC in China are still higher than those in other countries, suggesting that HCC poses a considerable threat to human life and health.^2^

LLR and RFA are two important methods for radical treatment of primary HCC. Compared with open liver resection, laparoscopic hepatectomy has the advantages of less trauma, faster postoperative recovery, and shorter hospital stay and has been widely used in the clinical treatment of HCC.^3^ RFA can achieve the same therapeutic effect as hepatic resection.^4^, ^5^, ^6^

The caudate lobe of the liver is adjacent to the surrounding ducts. Surgical treatment of caudate lobe HCC presents great technical challenges because of its special anatomical location. Previously, the caudate lobe was considered a forbidden area for LLR. With the advancements in laparoscopic technology, laparoscopic caudate lobe resection has become safe and feasible.^7^, ^8^ Studies have shown that the short‐term clinical efficacy of laparoscopic caudate lobectomy is better than that of open surgery, and its long‐term clinical efficacy is comparable with that of open surgery, with similar postoperative tumor‐free and overall survival.^9^ With improvements in RFA technology, RFA of special parts of the liver, including caudate lobe liver cancer, has achieved satisfactory clinical results.^10^ RFA and LLR have become important methods for treating caudate‐lobe HCC. However, few studies have compared the efficacies of LLR and RFA in the treatment of caudate lobe HCC. This study aimed to investigate the clinical efficacy of LLR versus RFA in the treatment of caudate lobe HCC.

## 2 MATERIALS AND METHODS

### 2.1 Patients

**FIGURE 1:**
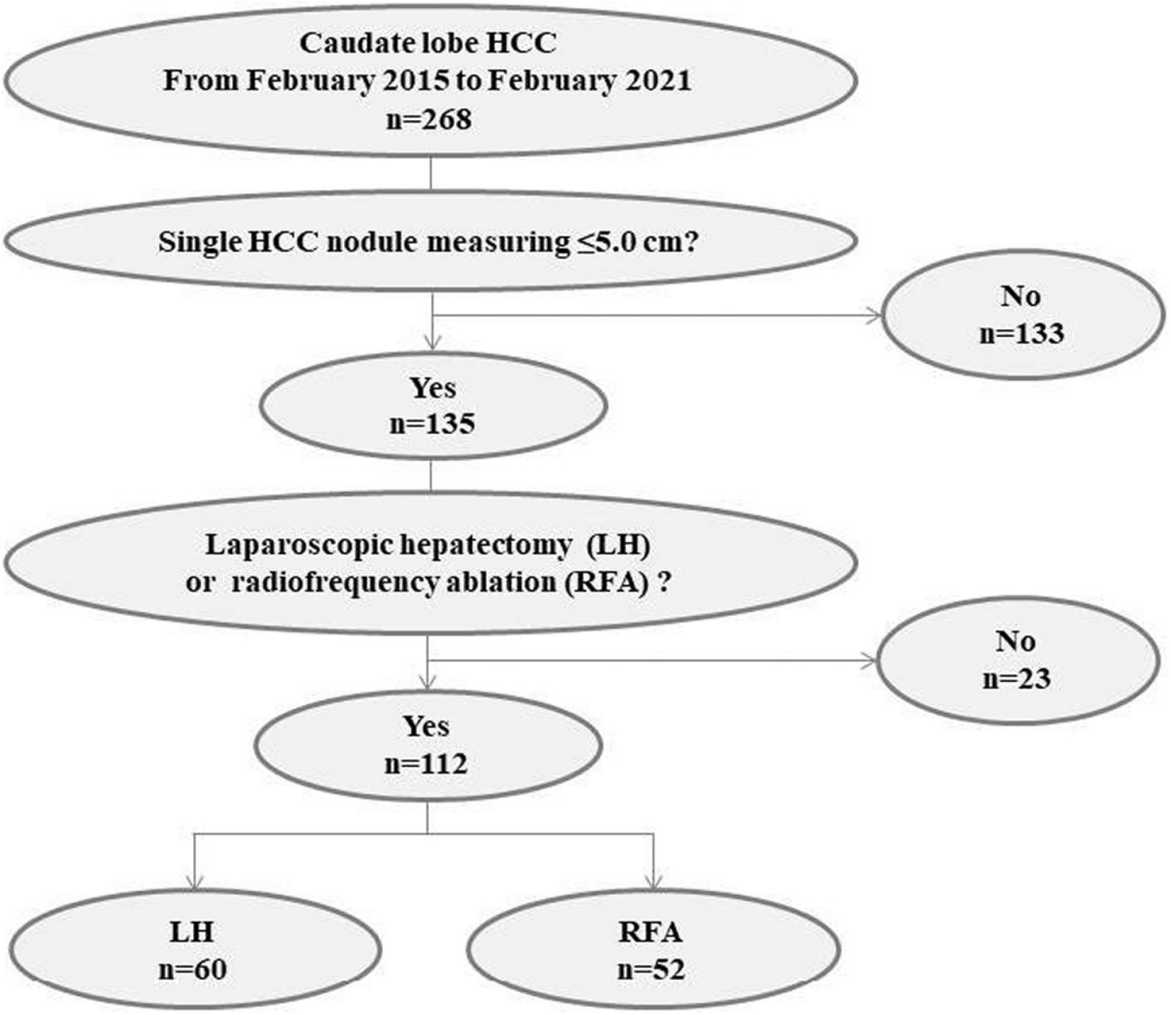
Flow chart of the whole study population.

This study included patients with HCC located in the caudate lobe who underwent LLR or RFA at the Affiliated Cancer Hospital of Zhengzhou University, Tianjin Medical University Cancer Hospital, and Chinese Academy of Medical Sciences‐affiliated Cancer Hospital between February 2015 and February 2021. This retrospective study was approved by the IRB of the above hospitals (approval number: 2023‐KY‐0015). Written informed consent was obtained from the IRB. The inclusion criteria were (1) single caudate lobe HCC nodule ≤5.0 cm confirmed by biopsy pathology or at least two enhanced image tests^11^; (2) the absence of intrahepatic or extrahepatic metastases; (3) the absence of vascular invasion; (4) Child–Pugh A/B score; (5) normal or prolonged prothrombin time <3 s; and (6) detected for the first time and treated with LLR or RFA. The exclusion criteria were as follows: (1) Non‐caudate‐lobe HCC; (2) multiple or single HCC nodules measuring >5 cm; (3) vascular invasion or extrahepatic metastases; and (4) the presence of other malignant tumors. A total of 112 patients met the inclusion criteria (Figure 1). Based on the different treatment methods, the patients were divided into LLR and RFA groups.

### 2.2 LLR procedure

Surgery was performed by surgeons with more than 10 years of experience in LLR. Intraoperatively, low central venous pressure anesthesia was used. Intraoperative ultrasonography was used to confirm the tumor site, size, and boundaries. The resection margin was approximately 1.0 cm away from the tumor boundary. If the tumor compressed the inferior vena cava or portal vein, it was stripped away from the surrounding pipelines to avoid pseudocapsule rupture. The vessel and bile duct were closed with a Hemolock clip and separated from the tumor. Blood from small blood vessels or wounds was coagulated using bipolar forceps. The Pringle method was applied to block blood flow into the liver if necessary; each block lasted 10 min, and the interval was 5 min. Depending on the location of the tumor in the caudate lobe, different surgical approaches were performed as follows^12^, ^13^, ^14^, ^15^: (1) For a tumor located in the Spiegel lobe, the laparoscopic left approach was used to perform tumor resection, the ligaments around the left lateral hepatic lobe were completely dissected with an ultrasonic scalpel, and the first hepatic hilum blocking band was pulled to the right to fully expose the Spiegel lobe. The short hepatic vein behind the caudate lobe was double‐clipped with Hemolock clips and dissected until the Spiegel's lobe tumor was completely removed. (2) For tumors located in the caudate process, the right laparoscopic approach was used for resection. The right perihepatic ligament was severed with an ultrasonic scalpel. The retrohepatic adrenal gland, and the inferior vena cava were exposed, and the first hepatic hilum blocking band was pulled to the left to fully expose the caudate process. The dissection of the hepatic parenchyma, anatomy of the short hepatic vein, and handling methods of the encountered intrahepatic vasculature are described above. (3) For tumors located in the paracaval portion of the caudate lobe or involving the entire caudate lobe, a combined laparoscopic right and left approaches was performed. A median approach was adopted to split the liver to achieve complete tumor resection or total caudate lobectomy, if necessary.

### 2.3 RFA procedure

A Samsung ACCUVIX A30 color Doppler ultrasound diagnostic instrument was used with an abdominal probe frequency of 3.5 MHz, and a detection depth of 20 cm. The RFA device used was a cold‐cycle RFA system, and the RFA needle was an LDDJS3‐0200250A (Lead Electron Corporation, Mianyang, China). The maximum output power is 200 W. A cold‐cycle single‐motor ablation mode was adopted for the ablation electrode needle. The length of the exposed electrode at the front was dynamically adjusted according to the specific tumor size (0–4 cm), and the output power and ablation time were adjusted according to the exposed length of the electrode front. Percutaneous RFA was performed on all patients. All patients were treated with conscious analgesia and sedation (flurbiprofen axetil 50 mg and dezocine 5 mg intravenously, 20 min before RFA) under ultrasound guidance. Hepatobiliary surgeons with more than 10 years of experience in RFA performed the RFA treatment. Trained radiologists performed ultrasound guidance to avoid important structures and organs percutaneously, and the radiofrequency electrode needle was inserted into the caudate. Three puncture approaches were used to insert the RF electrode needle according to the location and size of the tumor: the left‐side approach, right‐side approach, and combined left–right approach.^16^ The left‐side approach with the RF electrode needle through the liver space between the Glisson sheaths of Segments 2 and 3 penetrated the lesser omentum, and the RF electrode needle exited the liver parenchyma once. The right‐side approach passed through the right lobe of the liver but avoided the Glisson's sheath and right hepatic vein. For some lesions, a combined left–right approach with needle insertion was used for ablation therapy. The ablation was performed in automatic mode, and each ablation time was 8–12 min. Ultrasound was used for immediate evaluation during the ablation process. Multiple overlapping ablations were performed to obtain a sufficient ablation area when necessary. The ablation margins for all tumors, except subcapsular and closed to vital ducts, were planned to be at least 0.5 cm beyond the tumor boundaries. The ablation process ended when the hyperechoic region produced by RFA was sufficiently large to cover the entire tumor and surrounding normal liver tissue to obtain sufficient ablation margins on ultrasound. Contrast‐enhanced ultrasonography was performed. If there was significant enhancement in the arterial phase of the ablation area and regression in the equilibrium phase after angiography, the ablation was considered incomplete, and secondary ablation was performed directly.

### 2.4 Follow‐up

The last follow‐up date was April 16, 2022. The follow‐up time was defined as the time interval between the date of surgery or ablation and the date of death or last follow‐up. Patients were followed up monthly for the first 3 months after treatment, every 3 months from 3 months to 2 years, and every 6 months after 2 years. Follow‐up protocols included alpha‐fetoprotein (AFP) testing, chest CT, abdominal ultrasonography, contrast‐enhanced computed tomography, and contrast‐enhanced CT or imaging. Aggressive treatment was administered to patients with recurrence or metastasis; resurgical resection and ablation were the first choices for patients with a single lesion or fewer than three lesions with a diameter of less than 3.0 cm. Other non‐curative treatments include orally targeted drugs, lenvatinib, sorafenib, TACE, and immunotherapy.

### 2.5 Statistical analysis

All statistical analyses were performed using the Statistical Package for the Social Sciences (SPSS) version 27.0 (IBM, USA) and R version 3.6.1. Quantitative data following a normal distribution were expressed as mean ± standard deviation and compared using the Mann–Whitney *U*‐test or *t*‐test. Qualitative variables between groups were compared using the chi‐squared test, adjusted chi‐squared test, or Fisher's exact test, and cumulative OS was estimated using the Kaplan–Meier method. Factors affecting RFS and OS were determined using a Cox proportional hazards model. All *p*‐value calculations were two‐sided, and *p* < 0.05 was considered statistically significant.

PSM was performed to generate a propensity score for each patient using binary logistic regression analysis (caliper value, 0.10). The covariates entered into the model included sex, age, tumor site, tumor size, total bilirubin (TBIL), alanine aminotransferase (ALT), aspartate aminotransferase (AST), albumin (ALB), prothrombin time (PT), AFP, hepatitis virus, and Child–Pugh score. Subsequently, a one‐to‐one match between the LLR and RFA groups was obtained using nearest neighbor matching. Additionally, penalties were added when the propensity scores differed by more than 0.2 from the standard deviation. The matched data between the two groups were analyzed using conditional logistic regression.

### 2.6 Definitions

Local tumor recurrence (LTR) was defined as the appearance of tumor lesions at the margins of the surgical zones after LLR or RFA.

Overall survival (OS) was measured from the date of the initial treatment to the date of the last follow‐up or death.

Recurrence‐free survival (RFS) was measured from the date of the initial treatment to the date of recurrence, metastasis, or last follow‐up.

The Clavien–Dindo classification was used to grade postoperative complications.^17^, ^18^

## 3 RESULTS

### 3.1 Patient's baseline characteristics

Baseline characteristics of patients who received RFA (*n* = 52) and LLR (*n* = 60) were compared (Table 1 **)**. There were no differences in sex (*p* = 0.380), tumor location (*p* = 0.267), TBIL (*p* = 0.561), ALT (*p* = 0.860), AST (*p* = 0.146), ALB (*p* = 0.335), PT (*p* = 0.477), AFP positivity (>100 ng/mL) (*p* = 0.984), or Child–Pugh grade (*p* = 0.570) between the two groups. Patients in the LLR group were older (*p* = 0.025) and had a larger tumor size (*p* = 0.017) than those in the RFA group.

**TABLE 1:** Baseline characteristics of whole cohort and PSM cohort analyses.

Characteristics	Whole cohort (*n* = 112)		*p*	Matched cohort (*n* = 62)		*p*
	RFA group (*n* = 52)	LLR group (*n* = 60)		RFA group (*n* = 31)	LLR group (*n* = 31)	
Age (years)	52.6 ± 9.3	56.8 ± 10.2	0.025^c^	55.0 ± 9.7	55.0 ± 8.7	1.000^c^
Sex ratio (M:F)	37:15	38:22	0.380^a^	20:11	22:9	0.587^a^
Tumor location			0.267^a^			0.895^a^
S, *n* (%)	33(63.5)	46(76.7)		24(77.4)	23(74.2)	
P, *n* (%)	10(19.2)	6(10.0)		2(6.5)	3(9.7)	
C, *n* (%)	9(17.3)	8(13.3)		5(16.1)	5(16.1)	
Tumor size (cm)	2.43 ± 0.89	2.84 ± 0.88	0.017^c^	2.58 ± 0.93	2.68 ± 0.86	0.652^c^
Child–Pugh (A/B)	46/6	55/5	0.570^a^	29/2	28/3	1.000^a^
TBIL (mg/dL)	14.15(10.3–42.0)	13.95(9.4–37.5)	0.561^c^	13.9(10.3–35.1)	14.8(9.4–37.5)	0.395^d^
ALB (g/L)	38.9 ± 3.0	39.4 ± 2.8	0.335^c^	39.2 ± 3.1	39.1 ± 2.9	0.929^c^
PT (s)	12.6 ± 1.3	12.8 ± 1.3	0.477^c^	12.6 ± 1.3	12.9 ± 1.3	0.397^c^
ALT (U/L)	41.1 ± 16.2	40.6 ± 11.7	0.860^c^	41.6 ± 15.9	38.7 ± 8.9	0.385^c^
AST (U/L)	36.7 ± 15.9	40.6 ± 11.1	0.146^c^	37.4 ± 17.3	37.2 ± 11.4	0.959^c^
AFP >100 (ng/mL), *n* (%)	45(86.5)	52(86.7)	0.984^a^	27(87.1)	27(87.1)	1.000 ^a^
Viral hepatitis, *n* (%)	48(92.3)	54(90.0)	0.924^b^	30(96.8)	27(87.1)	0.351^b^
Cirrhosis, *n* (%)	46(88.5)	52(86.7)	0.775^a^	28(90.3)	29(93.5)	1.000^b^

*Note*: Values in parentheses are percentages unless indicated otherwise; measurement data are expressed as the mean ± standard deviation or the median (range).

Abbreviations: AFP, α‐fetoprotein; ALB, albumin; ALT, alanine aminotransferase; AST, aspartate aminotransferase; C, caudate process; HBV, hepatitis B virus; HCV, hepatitis C virus; S, Spiegel lobe; P, paracaval portion; PT, prothrombin time; TBIL, total bilirubin.

^a^ Chi‐squared test.

^b^ Chi‐squared test with a continuity correction.

^c^ Student's *t‐*test.

^d^ Mann–Whitney *U*‐test.

### 3.2 Intraoperative and postoperative characteristics

Intraoperative and postoperative characteristics of the two groups were compared before and after PSM (Table 2). In the LLR group, 58 patients underwent laparoscopic partial caudate lobectomy and two patients underwent total caudate lobectomy. Among the patients who underwent partial caudate lobectomy, two underwent right hemihepatectomy and three underwent left hemihepatectomy. The average operation time was 174.7 ± 36.1 min, and the mean intraoperative blood loss was 439.2 ± 222.8 mL. Three patients received perioperative blood transfusions. After surgery, there were two cases of bile leakage, two cases of infection, one case of ascites, and one case of pleural effusion. No complications such as bleeding, fat liquefaction, delayed wound healing, or liver failure were observed. The median hospital stay was 6 (3–15) days. Postoperative pathology showed one case of positive margins and three cases of microvascular invasion. Patients with marginal involvement were treated with oral targeted drugs before discharge. During follow‐up, 2 patients were lost, 1 patient developed LTR, 26 patients relapsed, and 20 patients died. Among patients with recurrence, 5 underwent surgical resection, 8 underwent radiofrequency ablation, 3 underwent radiofrequency ablation combined with systemic therapy, 6 underwent TACE combined with systemic therapy, and 4 underwent systemic therapy.

**TABLE 2:** Intraoperative and postoperative characteristics of whole cohort and PSM cohort analyses.

Characteristics	Whole cohort (*n* = 112)		*p*	Matched cohort (*n* = 62)		*p*
	RFA group (*n* = 52)	LLR group (*n* = 60)		RFA group (*n* = 31)	LLR group (*n* = 31)	
Operative time (min)	22.5 (10–80)	174.7 ± 36.1	<0.001^d^	25 (10–80)	181.7 ± 34.5	<0.001^d^
Blood loss (mL)	10 (2–30)	439.2 ± 222.8	<0.001^d^	10 (2–20)	420.0 ± 226.4	<0.001^d^
Transfusion, *n* (%)	0	3 (5.0)	0.295^b^	0	2 (6.5)	0.472^b^
Postoperative hospital stays, days	1.5 (1–7)	6 (3–15)	<0.001^d^	1 (1–7)	6 (3–9)	<0.001^d^
Overall morbidity, *n* (%)	4 (7.7)	5 (8.3)	1.000^b^	4 (12.9)	1 (3.2)	0.351^b^
Bile leakage, *n* (%)	0	2 (3.3)	0.498^c^	0	0	–
Infection, *n* (%)	1 (1.9)	2 (3.3)	1.000^b^	1 (3.2)	0	1.000^c^
Ascites, *n* (%)	1 (1.9)	1 (1.7)	1.000^c^	1 (3.2)	1 (3.2)	1.000^c^
Pleural effusion, *n* (%)	0	1 (1.7)	1.000^c^	0	0	–
Bleeding, *n* (%)	1 (1.9)	0	0.464^d^	1 (3.2)	0	1.000^c^
Spontaneous peritonitis, *n* (%)	1 (1.9)	0	0.464^c^	1 (3.2)	0	1.000^c^
Clavien‐Dindo grade I–II, *n* (%)	4 (7.7)	4 (6.7)	1.000^b^	4 (12.9)	1 (3.1)	0.351^b^
Clavien‐Dindo grade III–IV, *n* (%)	0	1 (1.7)	1.000^c^	0	0	–
Perioperative mortality, *n* (%)	0	0	–	0	0	–
Local tumor recurrence, *n* (%)	5 (16.1)	1 (1.7)	0.149^b^	3 (9.7)	0	0.237^b^
Treatments after recurrence			0.612^a^			0.765^b^
Liver resection	11	5		8	4	
RFA	6	8		4	5	
RFA+ systemic therapy	7	3		5	3	
TACE+ systemic therapy	10	6		5	2	
Systemic therapy	6	4		4	4	

^a^ Chi‐squared test.

^b^ Chi‐squared test with a continuity correction.

^c^ Fisher exact test.

^d^ Mann–Whitney *U*‐test.

In the RFA group, the left‐sided approach was used in 34 patients, the right‐sided approach in 12, and the combined left–right approach in 4. The average operation time was 22.5 (10–80) min, and the intraoperative blood loss was minimal. None of the patients received blood transfusion. One day after RFA, 2 patients with residual ascites underwent a second RFA treatment immediately. Additionally, there were 7 cases of fever, 1 case of infection, 1 case of bleeding, 1 case of spontaneous peritonitis, and 1 case of ascites. No complications, such as bile leakage, fat liquefaction, skin burns, or liver failure were observed. The median hospital stay was 1.5 (1–7) days. During follow‐up, 3 patients were lost, 5 patients developed LTR, 40 patients relapsed, and 32 patients died. Among patients with recurrence, 11 underwent surgical resection, 6 underwent radiofrequency ablation, 7 underwent radiofrequency ablation combined with systemic therapy, 10 underwent TACE combined with systemic therapy, and 6 underwent systemic therapy.

### 3.3 Efficacy analysis

**FIGURE 2:**
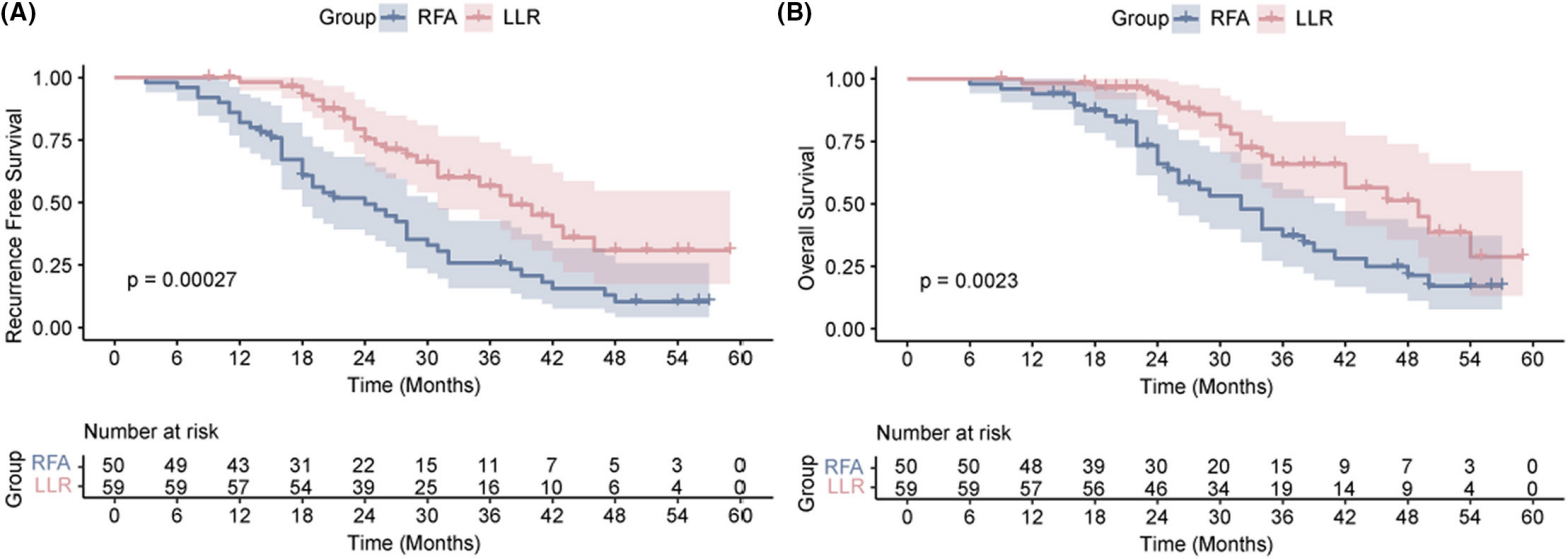
Recurrence‐free survival (RFS; A) and overall survival (OS; B) in LLR group and RFA group before PSM.

Before PSM analysis, the 1‐year, 2‐year, 3‐year RFS and OS rates in the LLR group were 98.2%, 75.5%, and 56.6% and 98.3%, 92.5%, and 65.8%, respectively. The 1‐year, 2‐year, 3‐year RFS and OS rates in the RFA group were 82%, 49.4%, 25.9% and 94%, 66.0%, and 37.2%, respectively. Regardless of the differences in age and tumor size between two groups, better OS and RFS were observed in the LLR group compared to RFA group using a logrank test (*p* = 0.0023 and *p* = 0.00027, respectively, Figure 2).

**FIGURE 3:**
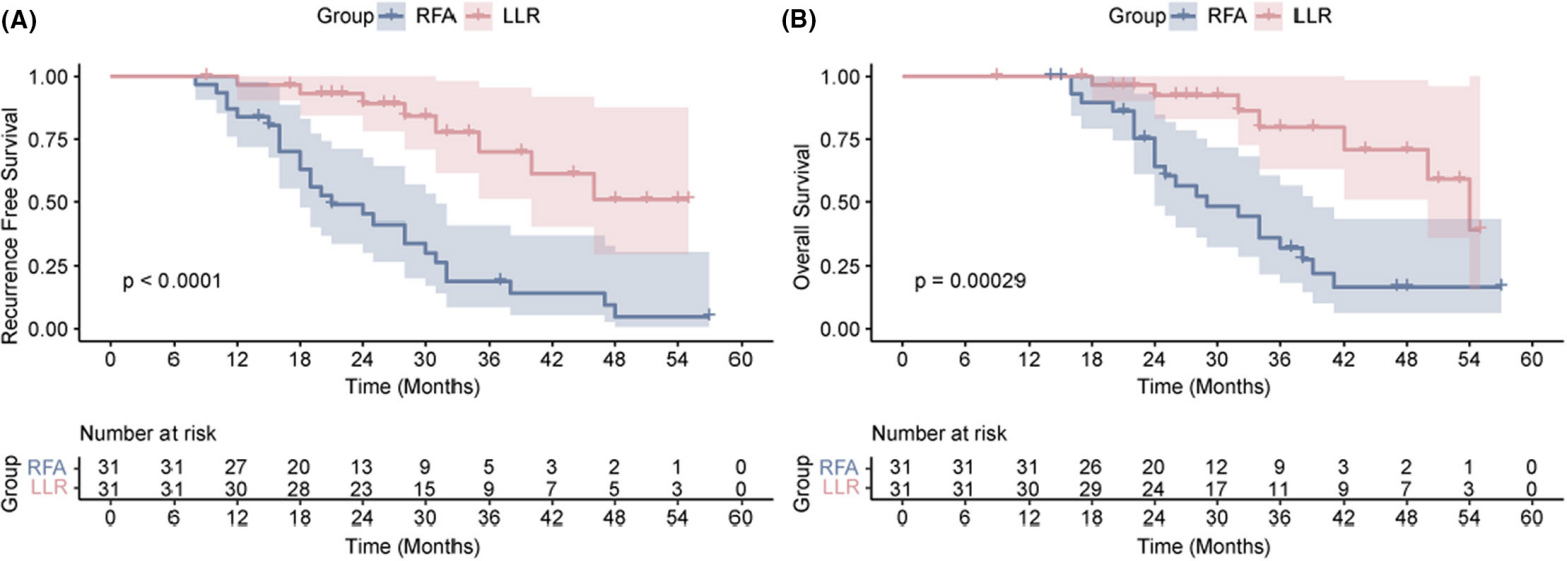
Recurrence‐free survival (RFS; A) and overall survival (OS; B) in LLR group and RFA group after PSM.

After PSM, 62 patients (31 undergoing LLR and 31 undergoing RFA) were screened for further analysis. The baseline characteristics of the two groups were comparable. The 1‐year, 2‐year, 3‐year RFS and OS rates in the LLR group were 96.7%, 89.2%, 70.0% and 100%, 92.5%, and 79.7%, respectively. The 1‐year, 2‐year, 3‐year RFS and OS rates in the RFA group were 83.9%, 45.2%, 18.8% and 100%, 64.1%, and 32.2%, respectively. Better OS and RFS were observed in the LLR group (*p* = 0.00029 and *p* < 0.0001, respectively; Figure 3).

### 3.4 Univariate and multivariate analyses

The univariate and multivariate analyses of RFS and OS after PSM are presented in Table 3. In the univariate analysis, tumor size (hazard ratio [HR], 2.965; 95% confidence interval [95% CI], 1.463–6.010; *p* = 0.003), AFP positivity (HR, 3.297; 95% CI, 1.115–9.750; *p* = 0.031), and LLR treatment (HR, 0.199; 95% CI, 0.090–0.441; *p* < 0.001) were significantly associated with RFS. Multivariate analysis also showed that tumor size (HR, 3.056; 95% CI, 1.435–6.507; *p* = 0.004), AFP positivity (HR, 3.727; 95% CI, 1.203–11.547; *p* = 0.023), and LLR (HR, 0.145; 95% CI, 0.063–0.333; *p* < 0.001) were independent risk factors for RFS.

**TABLE 3:** Univariate and multivariate analysis of RFS and OS after PSM.

Characteristics		RFS				OS			
		Univariable analysis		Multivariable analysis		Univariable analysis		Multivariable analysis	
		Hazard ratio	*p*	Hazard ratio	*p*	Hazard ratio	*p*	Hazard ratio	*p*
Group	RFA	0.199 (0.090–0.441)	<0.001	0.145 (0.063–0.333)	<0.001	0.230 (0.096–0.551)	0.001	0.219 (0.081–0.592)	0.003
	LLR								
Age (years)		1.006 (0.970–1.043)	0.746			0.995 (0.957–1.033)	0.782		
Sex	Female	0.985 (0.488–1.987)	0.965			1.015 (0.466–2.213)	0.970		
	Male								
Viral hepatitis	No	0.980 (0.297–3.238)	0.974			0.766 (0.229–2.558)	0.665		
	Yes								
Cirrhosis	No	0.664 (0.200–2.202)	0.503			0.748 (0.174–3.208)	0.698		
	Yes								
Child–Pugh	A	1.528 (0.458–5.103)	0.491			1.098 (0.257–4.686)	0.900		
	B								
Max size (cm)	≤3	2.965 (1.463–6.010)	0.003	3.056 (1.435–6.507)	0.004	3.032 (1.402–6.554)	0.005	2.555 (1.109–5.883)	0.028
	>3								
Location	S	Reference				Reference			
	P	1.194 (0.355–4.014)	0.774			1.174 (0.346–3.981)	0.797		
	C	1.349 (0.579–3.148)	0.488			1.066 (0.399–2.853)	0.898		
AFP (ng/mL)	≤100	3.297 (1.115–9.750)	0.031	3.727 (1.203–11.547)	0.023	2.712 (0.903–8.148)	0.076		
	>100								
PT(S)		1.156 (0.886–1.508)	0.285			1.220 (0.909–1.638)	0.186		
ALT (U/L)		1.000 (0.971–1.030)	0.991			1.010 (0.979–1.043)	0.522		
AST (U/L)		0.993 (0.970–1.017)	0.575			0.990 (0.964–1.016)	0.441		
ALB (g/L)		0.966 (0.850–1.098)	0.595			0.972 (0.841–1.124)	0.703		
TBIL (μmol/L)		1.002 (0.948–1.060)	0.932			0.992 (0.930–1.057)	0.796		
Treatment after recurrence		1.238 (0.950–1.612)	0.114			1.391 (1.026–1.886)	0.033	1.417 (1.057–1.901)	0.020

Univariate analysis showed that LLR treatment (HR, 0.230; 95% CI, 0.096–0.551; *p* = 0.001), tumor size (HR, 3.032; 95% CI, 1.402–6.554; *p* = 0.005), and treatment after recurrence (HR, 1.391; 95% CI, 1.026–1.886; *p* = 0.033) were significantly related to OS. Multivariate analysis revealed that LLR treatment, tumor size, and treatment after recurrence were independent risk factors for OS.

## 4 DISCUSSION

The overall incidence of HCC in the hepatic caudate lobe is relatively low. In previous literature reports, the number of cases in clinical studies of caudate lobe HCC is small. Even if multicenter clinical studies are used, the numbers are still limited, and there are no randomized controlled clinical studies.^19^, ^20^, ^21^ Owing to its special anatomical location and proximity to important surrounding channels such as the vena cava, hepatic vein, portal vein, and bile duct, the surgical treatment of HCC at this location presents great technical challenges. The caudate lobe was previously regarded as a “forbidden area” for the surgical resection of liver tumors.

However, the innovation and development of surgical techniques have gradually overcome this issue. In recent decades, laparoscopic technology has advanced, and the indications for LLR have continued to expand. At present, laparoscopic caudate lobectomy is safe and feasible, and has become a routine procedure in laparoscopic surgery. Laparoscopic partial caudate lobectomy, total caudate lobectomy, and caudate lobectomy combined with other hepatic segments have been reported.^14^, ^22^, ^23^, ^24^ The magnification, unique viewing angle, and long‐rod operation effect of laparoscopy can facilitate the resection of the caudate lobe in the deep anatomical position and find fine channels for precise treatment to reduce bleeding caused by injury to important vessels. Studies have shown that compared with open caudate lobectomy, laparoscopic caudate lobectomy has the advantages of less intraoperative blood loss and faster postoperative recovery. The curative effect is similar to that of open surgery.^25^, ^26^ Another clinical study using PSM analysis found that the laparoscopic group had earlier initiation of eating and getting out of bed, shorter postoperative analgesia, shorter hospital stays, and most patients had tumor resection margins of >10 mm. The postoperative complications in the two groups were not statistically different.^27^

Several HCC diagnosis and treatment guidelines recommend RFA as a radical treatment for early‐stage liver cancer.^28^, ^29^, ^30^ However, RFA treatment of caudate lobe HCC was previously considered challenging and high‐risk because the percutaneous puncture route is narrow, and the tumor is surrounded by the IVC, portal vein, and bile duct. They are prone to serious complications and local tumor recurrence after RFA.^31^ With the advancement of precision medicine, studies have reported that percutaneous RFA treatment of hepatic caudate lobe tumors is safe and feasible, with a technical success rate of 100%, technical efficacy rate of 91.67%, and a local recurrence rate of only 8.33%. No serious postoperative complications occurred.^10^ A study comparing TACE and RFA for caudate lobe HCC in compliance with the Milan criteria showed that the OS and LTR in the RFA group were better than those in the TACE group, regardless of whether a propensity score analysis was performed.^32^ A comparative study on the efficacy of RFA for liver tumors at high‐risk sites (such as the caudate lobe) and low‐risk sites found no statistical differences in complete ablation rate, complications, and perioperative mortality.^33^ Therefore, RFA is a safe and effective method for treating caudate‐lobe HCC.

In clinical practice, percutaneous RFA is widely used because of its simplicity, easy adjustment of the needle insertion route in real time, and the choice of general or local anesthesia according to the patient's tolerance. Percutaneous RFA under US guidance is safe and feasible. The postoperative local recurrence rate is low, and the application of artificial ascites to protect the perihepatic organs can achieve satisfactory clinical results.^20^, ^34^ Percutaneous RFA of caudate lobe HCC can be divided into various approaches according to the different needle insertions, such as the right intercostal, anterior epigastric, left and right combined, and dorsal approach.^35^, ^36^ Compared with other parts of liver cancer, RFA of caudate lobe HCC has a higher local recurrence rate, and tumors larger than 2 cm may be an important risk factor for local recurrence.^21^ Multivariate analysis of RFA for caudate lobe HCC ≤3.0 cm showed that tumor location and tumor diameter were independent risk factors for local recurrence. Researchers have considered that the heat sink effect of RFA on blood vessels around the caudal lobe might increase local recurrence owing to insufficient margin.^34^

Several studies have demonstrated the clinical value of LLR and percutaneous RFA in the treatment of caudate lobe HCC. A comparison of the survival outcomes between the two treatments has not yet been conducted. Therefore, this study aimed to explore the differences in efficacy between the two treatments for caudate lobe HCC. The PSM method was adopted to balance bias in the clinical data. The results showed that the postoperative recovery of the RFA group was faster and the hospital stay was significantly shorter than that of the LLR group. There was no statistically significant difference in the incidence of postoperative complications or local tumor recurrence between the two groups. The 1‐year and 3‐year RFS rates in the LLR and RFA groups were 98.2% and 56.6%, and 82% and 25.9%, respectively. The 1‐year, 3‐year OS rates in the LLR and RFA groups were 98.3% and 65.8%, and 94% and 37.2%, respectively. The 1‐year and 3‐year RFS and OS rates of the two groups were similar to those reported in previous studies.^6^, ^37^ Better RFS and OS were observed in the LLR group than in the RFA group. Multivariate analyses showed that tumor size, AFP positivity, and LLR were independent risk factors for RFS, whereas tumor size, LLR, and treatment after recurrence were independent risk factors for OS. Therefore, considering the long‐term prognosis after the two treatments for hepatic caudate lobe HCC, we believe that LLR should be recommended as the preferred treatment for a single caudate lobe ≤5.0 cm HCC.

This study has the following limitations: (1) Due to the low overall incidence of hepatic caudate lobe HCC, the sample size was small. (2) This was a retrospective study, and although PSM was used, there was still bias in the clinical data. (3) Our findings show the optimal treatment method for single HCC nodules, but the best practice for multiple or recurrent HCC remains unclear. Further large‐scale surveys using multi‐institutional or national clinical databases are required to overcome these limitations.

## AUTHOR CONTRIBUTIONS

**Zhengzheng Wang:** Funding acquisition (lead); writing – original draft (lead). **Jingzhong Ouyang:** Data curation (lead); formal analysis (lead). **Binyang Jia:** Data curation (supporting); software (supporting). **Yanzhao Zhou:** Data curation (supporting); supervision (lead). Data curation (supporting). **Yi Yang:**Data curation (supporting). **Xiaoting Li:** Formal analysis (supporting); investigation (lead). **Qingjun Li:** Supervision (lead); writing – review and editing (supporting). **Jinxue Zhou:** Conceptualization (lead); project administration (lead); writing – review and editing (lead).

## FUNDING INFORMATION

The study was sponsored by Henan Provincial Medical Science and Technology Research Project (LLRGJ20220191); Key Scientific Research Project of Colleges and Universities in Henan Province (23A320033); Henan Provincial Science and Technology Project (232102311080).

## CONFLICT OF INTEREST STATEMENT

All the authors declare that there is no conflict of interest.

## ETHICS STATEMENT

The study was approved by the Ethics Committee of Affiliated Cancer Hospital of Zhengzhou University & Henan Cancer Hospital, Tianjin Medical University Cancer Hospital and Chinese Academy of Medical Sciences affiliated Cancer Hospital (approval no. 2023‐KY‐0015).

## PATIENT CONSENT FOR PUBLICATION

Not applicable.

## Data Availability

All data generated during this study are available from the corresponding author on reasonable request.
